# Structural characterisation of TNRC6A nuclear localisation signal in complex with importin-alpha

**DOI:** 10.1371/journal.pone.0183587

**Published:** 2017-08-24

**Authors:** Jessica J. Chaston, Alastair Gordon Stewart, Mary Christie

**Affiliations:** 1 Molecular, Structural and Computational Biology Division, The Victor Chang Cardiac Research Institute, Sydney, New South Wales, Australia; 2 St Vincent’s Clinical School, Faculty of Medicine, University of New South Wales, Sydney, New South Wales, Australia; National University of Singapore, SINGAPORE

## Abstract

The GW182/TNRC6 family of proteins are central scaffolds that link microRNA-associated Argonaute proteins to the cytoplasmic decay machinery for targeted mRNA degradation processes. Although nuclear roles for the GW182/TNRC6 proteins are unknown, recent reports have demonstrated nucleocytoplasmic shuttling activity that utilises the importin-α and importin-β transport receptors for nuclear translocation. Here we describe the structure of mouse importin-α in complex with the TNRC6A nuclear localisation signal peptide. We further show that the interactions observed between TNRC6A and importin-α are conserved between mouse and human complexes. Our results highlight the ability of monopartite cNLS sequences to maximise contacts at the importin-α major binding site, as well as regions outside the main binding cavities.

## Introduction

**Fig 1:**
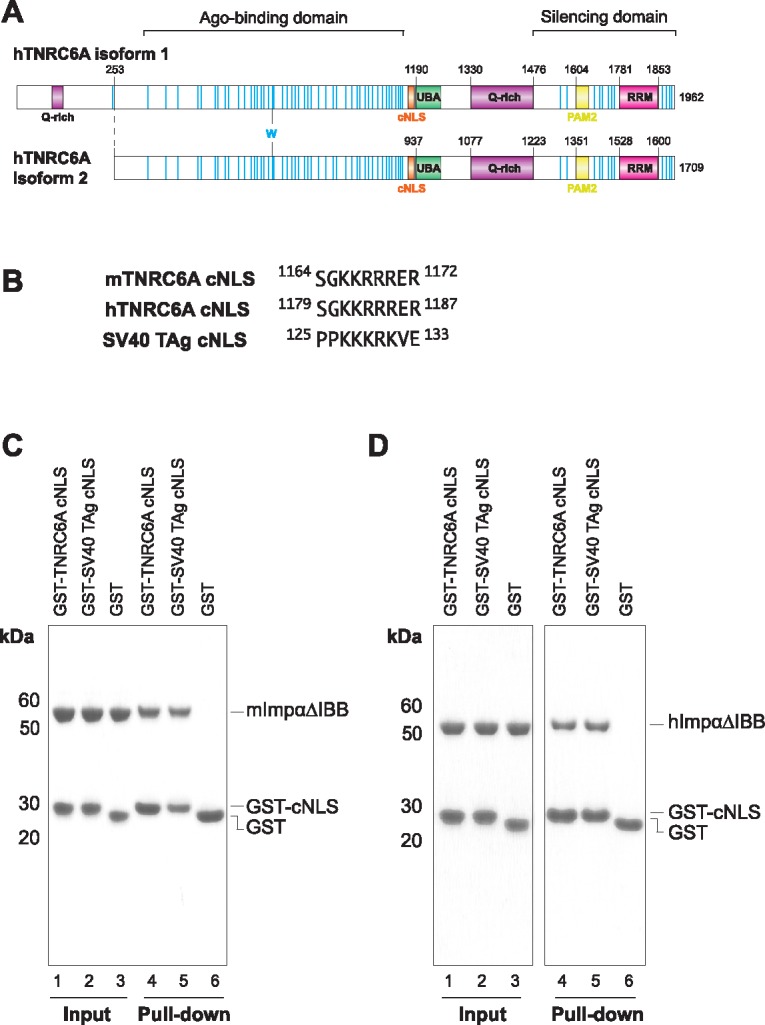
TNRC6A cNLS interaction with Impα is conserved between mouse and human proteins. (A) Schematic representation of hTNRC6A isoforms 1 and 2. TNRC6A cNLS is highlighted in orange, Trp residues are shown as blue lines. (B) Sequence of TNRC6A cNLS is strictly conserved between mouse TNRC6A (mTNRC6A) and hTNRC6A. The prototypic monopartite SV40 TAg cNLS is also shown. (C and D) *In vitro* pull-downs with GST-cNLSs and His-tagged mouse (C) and human (D) Impα1 proteins lacking the IBB domain (ΔIBB). GST and GST-SV40 TAg cNLS served as the negative and positive controls, respectively.

MicroRNAs (miRNAs) are master regulators of post-transcriptional gene expression, playing an important role in myriad cellular processes. miRNAs are loaded onto Argonaute (Ago) proteins to form the core of miRNA-induced silencing complexes (miRISCs), and exert their function through base complementarity, predominantly at 3’ untranslated regions (UTRs) of target mRNAs [1–3]. Plant miRNAs often display full base-pairing interactions with target sequences allowing direct cleavage of mRNAs by catalytically active Ago proteins [4]. By contrast, the majority of human miRNAs display only partial complementarity with mRNA targets requiring the association of a GW182/TNRC6 family member with one of four Ago proteins (termed Ago1-4) to trigger cytoplasmic mRNA degradation [5–7]. The GW182/TNRC6 family proteins are characterized by an abundance of (G/S/T)W(G/S/T) motifs (termed GW repeats) that are located throughout the length of the protein (Fig 1A); while the N-terminal GW motifs confer binding to Ago proteins [5, 8, 9], the C-terminal GW repeats are important for the recruitment of the PAN2-PAN3 and CCR4-NOT deadenylase complexes [10–15] for poly(A) tail removal, a process termed deadenylation. As deadenylation represses translation and can trigger irreversible mRNA degradation, the GW182/TNRC6 protein family are essential scaffolds that link miRISCs to the decay machinery.

There are three GW182 paralogs in vertebrates, namely, TNRC6A (also known as GW182), TNRC6B and TNRC6C, which share a characteristic architecture of a central ubiquitin associated domain (UBA) domain and a C-terminal RNA recognition motif (RRM) that are flanked by long stretches of sequences of intrinsic disorder (Fig 1A) [16]. As well as recruiting the deadenylase complexes, the GW182 C-terminus harbours a PAM2 motif that confers binding to the poly(A)-binding protein (PABP) [17–20], and a glutamine-rich (Q-rich) region that contributes to the localisation of the protein to cytoplasmic foci termed P-bodies [5, 21]. The C-terminal region of GW182/TNRC6 is termed the silencing domain, as this fragment is sufficient to induce silencing when tethered to an mRNA reporter [5, 21, 22] thus coordinating the downstream steps of miRNA-mediated mRNA decay.

Although miRNA-mediated silencing occurs in the cytoplasm, components of the miRISC have been detected in mammalian cell nuclei [23–27]. Recent reports have furthermore suggested nuclear roles for Ago including alternative splicing, as well as transcriptional silencing of nuclear RNAs [28–31]. Fluorescence microscopy analyses have demonstrated TNRC6 proteins also shuttle between nuclear and cytoplasmic compartments [32–34], consistent with the identification of a nuclear localisation signal (NLS) and nuclear export signal (NES) within the central region of TNRC6A [35]. Notably, immunostaining and cell fractionation analyses have demonstrated strong nuclear localisation of TNRC6A in some cancer cell lines [36, 37]. However, the nuclear function of TNRC6 and the importance of its nuclear import remain unclear. While reports suggest independent mechanisms for nuclear trafficking of Ago and TNRC6 proteins, the cytoplasmic levels of both proteins appear to be dependent on the import of the respective interaction partner, as well as mRNA abundance in the cytoplasm [33–35].

The majority of NLS containing proteins are termed classical NLSs (cNLSs), utilising the importin-alpha:importin-beta (Impα:Impβ) heterodimer for translocation into the nucleus [38]. Impα acts as an adaptor that binds cNLS cargo on the surface of its armadillo (ARM) repeat domain, while interacting with transport-competent Impβ proteins through an N-terminal Impβ-binding (IBB) domain [38–40]. cNLSs are short linear motifs, rich in basic amino acids. Monopartite cNLSs are comprised of one basic cluster, typified by the SV40 large T antigen (SV40 TAg) cNLS (^126^PKKKRKV^132^; basic cluster underlined), while bipartite cNLSs contain two basic clusters separated by a 10–12 residue linker, exemplified by the *Xenopus laevis* Nucleoplasmin cNLS (^155^KRPAATKKAGQAKKKK^170^; basic clusters underlined).

Impα is comprised of ten ARM repeats, which form an open solenoid structure [41, 42]. The inner surface of the Impα superhelix forms the cNLS binding groove, characterised by two binding regions termed the major and minor binding sites. Monopartite cNLSs bind preferentially to the Impα major binding site, located in ARM repeats 2–4. Bipartite cNLSs simultaneously interact with both Impα binding surfaces, with the N-terminal basic cluster located at the minor binding site (ARM repeats 6–8), and the basic residues of the C-terminal cluster contacting the major binding site [39]. Each of the Impα binding sites are comprised of a number of binding cavities that accommodate the basic side chains of the cNLS cluster; the minor binding site pockets are designated P1’, P2’ etc., while the cavities in the major binding site are termed P1, P2 etc. There are seven Impα paralogs in human and six in mouse, though their structure and cNLS binding determinants are highly conserved [39].

While a cNLS for human TNRC6A (hTNRC6A) has been described [35], this region is absent in TNRC6B and TNRC6C paralogs. However, a recent report has demonstrated that all three TNRC6 proteins can bind directly to Impβ [33]. In addition this work revealed that the presence of Ago2 reduced the binding of TNRC6A to Impα and Impβ, suggesting TNRC6A interaction with nuclear import receptors and Ago2 are mutually exclusive [33]. How Ago2 modulates TNRC6A binding to Impα and Impβ is unknown, though the close proximity of the Ago2 binding region suggests potential steric hindrance. To gain molecular insights into TNRC6A localisation, we have determined the structure of Impα in complex with the TNRC6A cNLS. The structure reveals that TNRC6A binds to the major binding site of Impα in a manner similar to canonical cNLSs.

## Materials and methods

### Protein preparation

GST-TNRC6A cNLS (residues 1164–1172 of mouse TNRC6A corresponding to residues 1179–1187 of the human protein; numbering according to hTNRC6A isoform 1) and mutants were cloned into pGEX-6P (GE Healthcare) using overlapping oligonucleotides. GST-cNLS fusions were expressed in BL21(DE3) (NEB) cells overnight at 20°C following isopropyl β-D-1-thiogalactopyranoside (IPTG) induction. Mouse Impα1 lacking the IBB domain (mImpα1ΔIBB; residues 70–529; also known as KPNA2) was expressed and purified as previously reported [43]. mImpα1ΔIBB mutants were obtained by site-directed mutagenesis and expressed and purified using similar methods as the wild-type protein. Human Impα1ΔIBB (hImpα1ΔIBB) was expressed in BL21(DE3) (NEB) cells in LB media at 25°C for 5 hours after induction with IPTG, and purified using similar techniques. The TNRC6 cNLS: mImpα1ΔIBB complex was isolated as described previously [44].

### Crystallisation, data collection and structure determination

Crystals of the mImpαΔIBB-TNRC6A cNLS were obtained in sodium citrate at pH 7.0 in the presence of 10 mM dithiothreitol (DTT), and cryoprotected with reservoir solution supplemented with 25% glycerol. Data was collected at the MX2 beamline at the Australian Synchrotron at a temperature of 100K using a wavelength of 0.95 Å. Data were integrated using iMOSFLM [45] and scaled with AIMLESS [46]. The structure was solved by rigid body refinement using the coordinates for mImpα1ΔIBB (PDB ID 1PJN [47]) with cNLS and water removed using REFMAC5 [48]. Simulated annealing was performed in Phenix [49] in an attempt to remove model bias, and manual model building of mImpα1ΔIBB was performed in real space using Coot [50]. Strong difference density corresponding to two peptides were observed and TNRC6A cNLS residues were built using Coot. The final model had excellent geometry and a MolProbity [51] score of 1.14.

### *In vitro* pull-downs

GST pull-down assays were performed in binding buffer (20 mM Tris pH7.5, 125 mM NaCl, 1 mM DTT). Glutathione resin (Pierce) was incubated with 100 μg of purified GST-cNLS or GST, and 250 μg of ImpA or 500 μg of ImpB at 4°C for 1 hr. Unbound protein was removed by washing beads 5 times in 500 μL binding buffer, and the bound proteins were eluted in binding buffer supplemented with 10 mM glutathione. The samples were analysed using SDS-PAGE, input lanes correspond to 1% of incubated samples and pull-down lanes correspond to 8% of eluted protein.

### Plate binding assay

Solid phase binding assays were performed as previously described [44, 52–54], with cross-linking omitted. MaxiSorp plates (Nunc) were coated with 25 nM of GST-cNLS or GST in coating buffer (PBS supplemented with 0.2 mM phenylmethylsulfonyl fluoride (PMSF) and 2 mM DTT). Plates were blocked overnight in coating buffer supplemented with 3% BSA and 0.1% Tween 20. Serial dilutions of S-tagged mImpα1ΔIBB were added and incubated for 1 hr at 4°C. Plates were washed 3 times by immersion in coating buffer supplemented with 0.1% Tween 20, and detected with S-protein-HRP conjugate (Novagen) using 3,3’, 5, 5’-tetramethylbenzidine as a substrate. The reaction was stopped by the addition of 1 M HCl, and the signal read at 450 nm using a PHERAstar plate reader (BMG LABTECH).

## Results and discussion

hTNRC6A cNLS (^1179^SGKKRRRER^1187^; numbering according to isoform 1 and corresponds to ^926^SGKKRRRER^934^ in isoform 2; Fig 1A) is located between the Ago-binding and UBA regions of the protein [35]. This motif is strictly conserved between mouse and humans (Fig 1B), is present in bats and hedgehogs, but appears to be absent in fish and insects. Previous reports have demonstrated that this region (comprising residues 1178–1187 and 1163–1172 in the human and mouse proteins, respectively) is sufficient to induce the nuclear localisation of a GFP reporter in HeLa cells, while mutation of the basic cluster prevented nuclear accumulation of identical cargo [35]. Although *in vitro* GST pull-downs have shown a direct interaction between TNRC6A and human Impα, these assays were performed in the context of the full-length protein and in the presence of Impβ, which forms additional contacts with TNRC6A outside of the described cNLS region [33]. We therefore wanted to assess if this motif was sufficient to directly bind Impα in the absence of Impβ.

We utilised a construct of mImpα1 that lacked the N-terminal IBB domain (mImpα1ΔIBB) as this region of the protein competes for cNLS binding through autoinhibition when Impβ is absent [40, 42, 55]. This construct thus mimics the conformation of Impα when in complex with Impβ. The analysis demonstrates that GST-tagged TNRC6A cNLS is sufficient to interact with both mImpα1ΔIBB (Fig 1C) and hImpα1ΔIBB (Fig 1D) proteins. By contrast, this region was not sufficient to pull-down mouse or human Impβ under similar conditions (S1 Fig). This is consistent with a previous mutational analysis which showed that Ala substitution of the four Arg residues (termed NLS-mut [35]) did not disrupt Impβ binding in the context of the full-length TNRC6A protein [33]. We therefore wanted to look at the Impα-TNRC6A cNLS interaction on a molecular scale.

**Fig 2:**
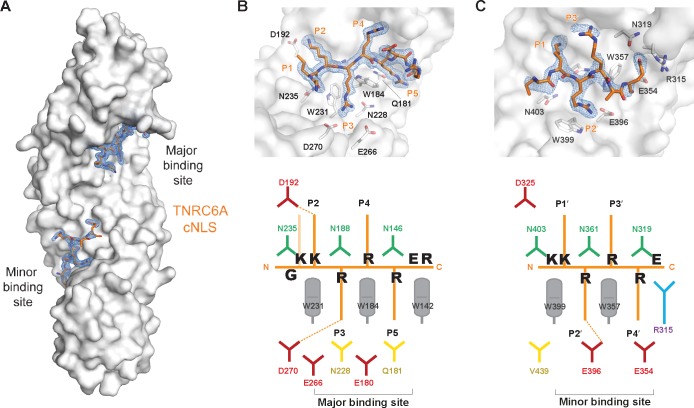
Structure of mImpαΔIBB in complex with TNRC6A cNLS. (A) Overall structure with mImpαΔIBB shown in surface representation (grey) and TNRC6CA cNLS residues in orange sticks. After rigid body refinement, addition of waters and real space adjustments of mImpαΔIBB, simulated annealing was performed and the calculated Fo-Fc map is shown as blue mesh contoured at 3σ. Close up view of the TNRC6A cNLS peptide at the mImpα (B) major and (C) minor binding sites. The Fo-Fc density as in (A) is overlaid. Schematic representation of the interactions observed in the crystal structure are shown below.

Orthorhombic crystals were obtained of mImpα1ΔIBB in complex with TNRC6A cNLS (residues 1164–1172 of the mouse protein corresponding to residues 1179–1187 of the human protein; Fig 1B) isomorphous to previously determined mImpα1ΔIBB:cNLS structures (Table 1). The data were refined to 1.9 Å resolution and display high structural similarity to previously determined mImpα-cNLS complexes (rmsd of 0.3 Å, 0.5 Å and 0.3 Å compared to mImpα bound to SV40 TAg (PDB ID 1EJL [56]), PepTM (PDB ID 3L3Q [57]) and Bimax 2 (PDB ID 3UKX [44]) structures, respectively. TNRC6A residues Gly1165-Arg1172 could be built unambiguously into strong difference density located at the mImpα1 major binding site (Fig 2A). The peptide binds in an extended conformation, with basic side chains interacting with the Impα binding pockets. These cavities are formed through a conserved Trp array on the concave surface of the Impα ARM repeats (Fig 2B and 2C), while conserved Asn residues interact with the cNLS main chain.

**Table 1:** Crystallographic data for mImpα1ΔIBB:TNRC6A cNLS peptide.

Data Collection	MX2 beamline Australian Synchrotron
Space group	P2_1_2_1_2_1_
Cell Dimensions a,b,c (Å)	78.79, 89.98, 99.28
Resolution (Å)	1.9
Rsym^#^	0.051 (0.609)
<I/σ(I)>	19.8 (2.8)
Completeness (%)	100 (100)
Redundancy	6.9 (7.0)
Observations	386 273 (24 884)
Unique reflections	56 325 (3573)
**Refinement**	
R_free_/ R_work_ (%)	0.152/ 0.182
Average B-factor (Å^2^)	41
R.m.s deviations from ideal values	
Bond lengths (Å)	0.017
Bond angles (°)	1.244
Ramachandaran plot (%)	
Favoured	98.9
Allowed	1.1
Forbidden	0

^#^values in parentheses correspond to highest resolution shell

**Fig 3:**
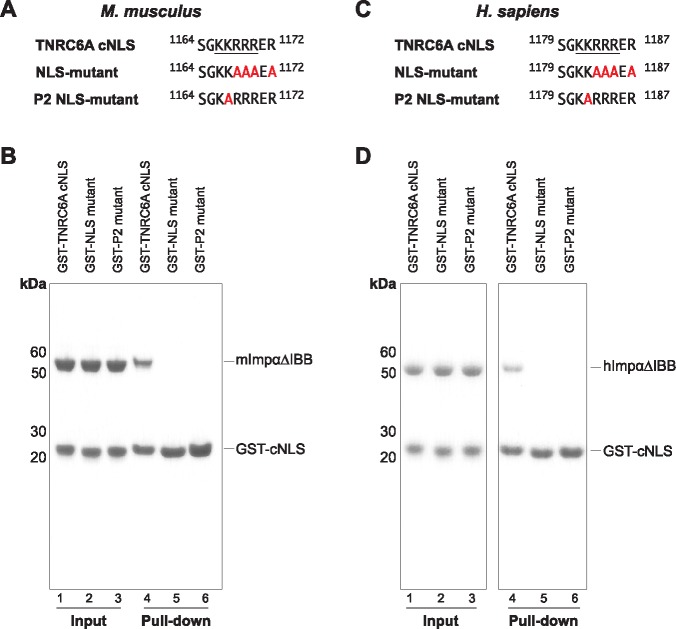
Mutation of the TNRC6A cNLS abolishes Impα interaction. (A) Sequences of mouse TNRC6A wildtype and mutant cNLSs. NLS mutant corresponds to substitutions previously described [35]. P2 mutant corresponds to an Ala substitution of Lys1167 (Lys1182 in the human protein), which binds to the P2 pocket of Impα major binding site. mTNRC6A cNLS residues located at the P1-P5 cavities of mImpα1ΔIBB are underlined. (B) GST pull-downs using purified GST-TNRC6A cNLSs and mImpα1ΔIBB. Wildtype TNRC6A cNLS served as the positive control. (C) Sequence of hTNRC6A WT and mutant cNLSs analogous to that used for the mouse protein in (A). The analogous cNLS motif that binds to the P1-P5 pockets of mImpα1ΔIBB is underlined. (D) GST pull-downs using purified GST-TNRC6A cNLSs and hImpα1ΔIBB.

The TNRC6A cNLS binds to the Impα surface such that all 5 basic side chains are located at positions P1-P5 of the major binding site, highly similar to the SV40 TAg monopartite cNLS (Fig 2B; Table 2). In this binding register, TNRC6A Lys1167 (Lys1182 in hTNRC6A) is termed the critical P2 lysine forming a salt bridge with Asp192 of mImpα1. The P2 lysine provides the greatest estimated free-energy contribution in both SV40 TAg and c-Myc cNLSs [58], and is almost strictly conserved in all Impα:cNLS structures determined to date [39]. Consistently, mutation of mTNRC6A Lys1167 to an Ala (termed P2 mutant; Fig 3A) completely abolished binding to mImpα1ΔIBB in pulldown assays (Fig 3B Lanes 3 and 6). Similar disruptions were observed between hTNRC6A cNLS mutants in binding the hImpα1ΔIBB (Fig 3C and 3D) suggesting the mode of binding is conserved between mouse and human complexes. This is further supported by contact analysis which reveals that all Impα residues that interface with TNRC6A cNLS are strictly conserved in hImpα1, and highly conserved in the other human paralogs (S2 Fig).

**Table 2:** Comparison of monopartite cNLS interactions with Impα major binding site.

cNLS	N-term	Major binding site pockets					C-term	PDB ID
		P1	P2	P3	P4	P5		
TNRC6A	G	K	*K*	R	R	R	ER	5UMZ; this study
SV40 TAg	PP	K	*K*	K	R	K	V	1EJL [56],1BK6* [41],4B8O^ [53],4RXH^^ [59]
PepTM	PF	K	*K*	K	R	R	EA	3L3Q [57]
c-Myc	PA	A	*K*	R	V	K	L	1EE4* [60]
dUTPase	AISP	S	*K*	R	A	R	PA	4MZ5 [61]
dUTPase S11E	AIEP	S	*K*	R	A	R	PA	4MZ6 [61]
Ku80	GPT	A	*K*	K	L	K	TE	3RZ9 [62]
TAF8	P	V	*K*	K	P	I	RR	4WV6** [63]
pUL56	AT	R	*K*	R	P	R	RA	5HUY [64]
pUL15	PP	K	*K*	R	A	K	VD	5HUW [64]
XPG1	SSS	L	*K*	R	K	R	LS	5EKF [65]

*, **, ^ and ^^denotes *S*. *cerevisiae*, human, *Oryza sativa* and *Neurospora crassa* proteins, respectively. All other structures correspond to mImpα1. Critical Lys residue that forms a salt bridge with Asp side chain at Impα P2 pocket is italicised.

**Fig 4:**
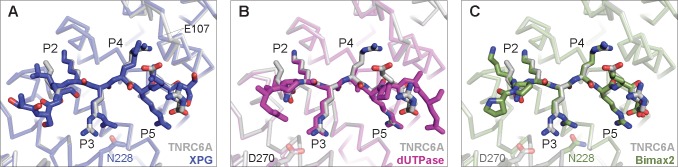
TNRC6A cNLS maximises interactions with the mImpαΔIBB major binding site cavities. In all panels, the mImpα1ΔIBB: TNRC6A cNLS complex is shown as grey ribbon and grey stick representation, respectively. (A) Comparison of cNLS binding at the mImpα P4 cavity. The mImpα1ΔIBB: XPG cNLS complex (PDB ID 5EKF [65]) is shown in blue. The mainchains of Arg106 and Glu107 (residue indicated) move to accommodate Arg binding at the P4 pocket in the case of TNC6A cNLS. (B and C) Comparison of cNLS binding at the mImpα P3 pocket. mImpα1ΔIBB: dUTPase S11E mutant complex (PDB ID 4MZ6 [61]) is shown in magenta (B); and mImpα1ΔIBB: Bimax 2 inhibitor complex (PDB ID 3UKX [44]) is shown in green (C). Arg side chains at the P3 position adopt different conformations to interact with Asp270 (TNRC6A and dUTPase S11E mutant) or Asn228 (Bimax2 inhibitor), which are highlighted as sticks.

The residues of TNRC6A cNLS at the P3-P5 pockets form H-bond interactions with the Impα surface. More specifically, Arg1169 at the P4 pocket contacts the mImpα mainchain in the loop between ARM repeats 1 and 2 (Arg106-Glu107) that causes a shift in the Cα backbone when compared to cNLS bound structures that contain smaller residues, including lysine, at that position (Fig 4A). The TNRC6A Arg1170 side chain is projected into the P5 cavity that is lined by Trp142 and Trp184, interacting with Gln181 at the end of the mImpα P5 pocket. At the same time, Arg1168 in the P3 position forms a salt-bridge with Asp270 on the mImpα surface. This interaction has previously been observed in other cNLS:Impα structures such as the dUTPase S11E phospho-mimic peptide when bound to mImpα1 [61] (Fig 4B) as well as the c-Myc peptide in complex with *Saccharomyces cerevisiae* Impα [60] (S3A Fig). This is in contrast to that observed in Bimax2 and XPG interactions with mImpα, which instead utilise basic side chains at the P3 position to H-bond with Asn228 (Fig 4C). In *S*. *cerevisiae*, Ala mutagenesis of c-Myc and SV40 TAg cNLSs suggested the preference for Arg over Lys at the P3 pocket [58]. However, *S*. *cerevisiae* Impα contain a threonine (Thr234) residue at the position analogous to Asn228 suggesting that longer Arg side chains may be able to form more favourable interactions with the Impα surface. Consistent with our structural observations, binding was disrupted when the four arginine residues of TNRC6A cNLS were substituted to Ala (NLS mutant; Fig 3). Notably, the side chains of Arg1172 at the very C-terminus of the cNLS nor of Lys1166 at the P1 position, do not specifically interact with the surface of mImpα1.

The basic cluster of the TNRC6A sequence is highly similar to the C-terminal region of the bipartite Bimax2 peptide inhibitor of Impα (S3B Fig), which was previously identified through activity-based profiling [66]. In addition, it resembles the PepTM sequence (Table 2) that was derived from a peptide library approach to identify high affinity monopartite cNLS sequences [57]. This suggests that the sequence of TNRC6A cNLS is highly similar to optimised high affinity peptides directed at the Impα major binding site. This is reflected in the apparent K_d_ between mImpα1ΔIBB and the monopartite cNLS peptides (apparent K_d_ values of 53.2 ± 14.9 and 75.62 ± 21.15 nM for PepTM [57] and TNRC6A cNLSs, respectively; Table 3).

**Table 3:** Dissociation constants for mImpα and cNLSs.

	K_d_ apparent (nM)
GST-SV40 TAg cNLS	12.48 ± 1.9
GST-TNRC6A cNLS	75.62 ± 21.1
GST-TNRC6A cNLS P3 mutant	1875 ± 254.6
GST-TNRC6A cNLS P5 mutant	1141 ± 219.6
GST-TNRC6A cNLS P3, P5 mutant	898.4 ± 104.0
GST-TNRC6A cNLS N-term mutant	18.99 ± 2.67

Assays were performed in duplicate; values represent the mean with standard error calculated by Prism (GraphPad) using one-site specific binding.

**Fig 5:**
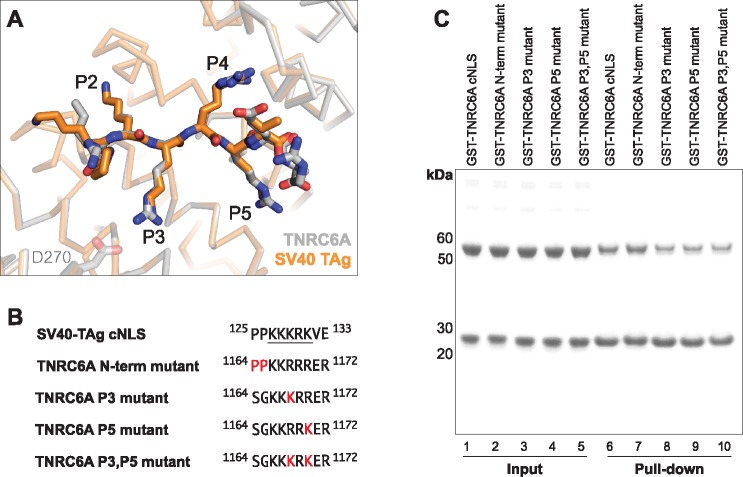
TNRC6A mutants that more closely resemble the SV40 TAg cNLS retain mImpΔIBB binding capacity. (A) Superposition of mImpα1ΔIBB: TNRC6A cNLS (grey ribbon and grey stick representation, respectively) and mImpα1ΔIBB in complex with SV40 TAg cNLS (shown in orange; PDB ID 1EJL [56]). The two cNLS basic clusters differ at positions P3 and P5 of the mImpα1ΔIBB binding pockets. (B) Sequences of the TNRC6A cNLS mutants that were generated to be more similar to the SV40 TAg cNLS motif. Residues mutated are highlighted in red. SV40 TAg cNLS residues located at the P1-P5 cavities of mImpα1ΔIBB are underlined. (C) GST pull-downs using purified GST-TNRC6A mutant cNLSs and mImpα1ΔIBB. Wildtype TNRC6A cNLS served as the positive control.

Structural comparison of mImpα1ΔIBB in complex with TNRC6A and SV40-TAg cNLSs reveals high structural similarity between the two monopartite peptides at the major binding site (Fig 5A). Both sequences contain five basic residues that bind to the P1-P5 pockets, differing at the P3 and P5 positions (Table 2). Despite the high sequence and structural similarity at the P1-P5 cavities, the SV40 TAg cNLS interacts with the mImpα1ΔIBB surface with a higher apparent K_d_ (Table 3). Outside of these binding positions, the SV40 TAg and TNRC6A cNLSs differ at the N- and C-terminal regions (Table 2). Notably, the SV40 TAg cNLS contains a ^124^Pro-Pro^125^ motif directly N-terminal to the monopartite basic cluster, which are the optimal residues at that region in bipartite cNLS sequences (S3B Fig) [66]. By contrast, TNRC6A cNLS contains a ^1164^Ser-Gly^1165^ motif (Fig 1B); Ser1164 did not appear to be ordered in our crystals suggesting that it does not form specific contacts with the mImpα1ΔIBB surface.

To determine the sequence element of the SV40 TAg cNLS that confers higher affinity binding, we generated TNRC6A cNLS mutants that more closely resembled the SV40 TAg sequence. To this end, residues at the N-terminus of the cNLS, or within the P3 and/ or P5 positions were substituted to mimic the SV40 TAg sequence (Fig 5B). The C-terminal residues were not altered as they do not make any specific H-bond or salt bridge interactions with mImpα1ΔIBB in either SV40 TAg or TNRC6A cNLS complex structure. All mutants generated retained the ability to interact with mImpα1ΔIBB in GST pull-down assays under the conditions tested (Fig 5C). Solid-phase binding experiments, however, revealed that the three P3 and/ or P5 pocket mutants had markedly reduced apparent K_d_ measurements compared to the wild type sequence (Table 3).

While it is well-established that the sequence determinants of monopartite cNLSs are more stringent than any polybasic cluster, the general consensus sequence for binding to the Impα P2-P5 pockets has been described as **K**(K/R)*X*(K/R) (where the P2 lysine is highlighted in bold, and *X* represents any amino acid) [39].Thus, all TNRC6A P3 and/ or P5 cavity mutants adhere to this general monopartite consensus sequence. However, the Arg1168Lys substitution at the P3 pocket would abrogate the salt bridge interaction observed between mImpα1ΔIBB Asp270 and wild type TNRC6A cNLS (Fig 5A); the Lys129 side chain of SV40 TAg cNLS located the P3 cavity makes no specific contacts with the mImpα surface. Mutation of Arg1170 to a lysine at the P5 position did not reduce binding to the same extent as the P3 TNRC6A mutant, consistent with the observation that both Lys and Arg cNLS residues mediate H-bonding interactions with mImpα at this position. As the basic cluster of the TNRC6A cNLS P3,P5 mutant is identical to that of the SV40 TAg motif (Fig 5B), our results imply that additional sequence determinants are required for high affinity binding to mImpα1ΔIBB. We note that solid phase binding assays, although commonly employed in the field [44, 52, 53, 57, 67, 68], are not as sensitive as other methodologies and are initiating binding kinetics measurements between the TNRC6A cNLS mutants and Impα to more accurately determine binding affinities.

A detailed mutational analysis of a template monopartite NLS sequence has been reported, where all possible 16 Lys and/or Arg combinations (SSGA####AG, where # represents either Lys or Arg) predicted to bind at the P2-P5 pockets, were tested for their nuclear import activity [69]. This analysis revealed that not all combinations of four consecutive Lys/ Arg residues are sufficient to induce complete nuclear accumulation of a fluorescent reporter. Complete nuclear accumulation was observed for a variant (SSGAKRRRAG, Lys/ Arg combination underlined) with a basic cluster similar to the wild type TNRC6A cNLS sequence [69]. However, the sequences that induced partial nuclear localisation generally agreed with the TNRC6A P3 and/or P5 mutants that had reduced binding to mImpα1ΔIBB compared to the wild type cNLS in our plate binding assays. Although the nuclear import activity of the generated P3 and/ or P5 pocket mutants have not been characterised, cNLSs with similar apparent K_d_ values have been shown to import fluorescent cargo into the nucleus of permeabilized human cells using *in vitro* nuclear translocation assays [53]. Further work is therefore required to establish whether the P3 and/or P5 mutants affect the translocation activity of TNRC6A.

Due to the five consecutive basic residues in the TNRC6A sequence, register staggering in the mImpα1ΔIBB binding pockets may be possible for the TNRC6A cNLS constructs (eg. Lys1166 binding at the P2 pocket instead of Lys1167), as previously observed in other cNLS peptides [44, 56, 65]. Indeed, it has previously been suggested that the lower affinity measured for the XPG2 peptide compared to the XPG1 peptide may be related to peptide staggering or multiple interaction modes of the cNLS, evidenced through ambiguous electron density for XPG2 peptide when bound to mImpα1ΔIBB [65]. While no indication for register staggering was observed in the crystal structure of wild type TNRC6A cNLS in complex with mImpα1ΔIBB, further studies are required to establish whether mutation of the basic cluster motif, such as that of the P3 and/or P5 mutants, may cause the TNRC6A cNLS to adopt alternative binding register modes in order to maximise favourable interactions at the major binding site pockets. Together, this suggests that the pattern of the TNRC6A cNLS basic cluster is optimal for mImpα1ΔIBB interaction in the context of the wild type sequences flanking these positively charged residues, and single or double conservative substitutions at the P3 and or P5 positions decrease *in vitro* binding to the Impα surface.

By contrast, the measured apparent K_d_ for the TNRC6A N-term mutant, whereby ^1164^Ser-Gly^1165^ was substituted to prolines (Fig 5B), was enhanced for mImpα1ΔIBB binding (Table 3) with an apparent K_d_ measurement similar to that observed for SV40 TAg cNLS. This is consistent with the observation that a Pro-Pro motif directly N-terminal to the P1 pocket is the optimal sequence for Impα binding in bipartite cNLSs (S3B Fig), and suggests that prolines may also be preferred to a Ser-Gly motif in the analogous region of monopartite cNLSs. The relative energy contributions of each residue of the SV40 TAg sequence (^126^PKKKRKV^132^) has been calculated based on the measured binding affinity between *S*. *cerevisiae* ImpαΔIBB and cNLS mutants generated by alanine scanning [58]. This analysis suggested that the energy contribution of Pro126 is modest at approximately 1/5 of that observed for the P2 Lys127 residue. As Pro125 of the SV40 TAg cNLS was not considered in this study, the contribution of this residue, in both SV40 TAg and TNRC6A N-term mutant cNLSs, to Impα interaction is unclear. Nonetheless, our binding assay measurements are consistent with previous reports that demonstrate that additional contacts outside of the P1-P5 pockets can contribute to cNLS interaction with Impα to enhance binding affinity [66, 69, 70]. Together with our observations for the P3 and/or P5 TNRC6A mutants, our data indicates that both the basic pattern of the cNLS cluster, as well as the flanking N-terminal motifs can modulate the interaction between monopartite cNLS peptides and mImpαΔIBB.

At the minor binding-site, TNRC6A cNLS residues Lys1166-Glu1171 could be built into difference density maps located at mImpα1 ARM repeats 6–8. The TNRC6A cNLS binds with the typical Lys-Arg motif located at the P1’ and P2’ positions at the minor binding site as seen in other cNLS sequences (Fig 2C and Table 4). TNRC6A Arg1168 and mImpα1 Glu396 form an ion pair that is highly conserved in other cNLS interactions with Impα. The cNLS residues in the minor binding site cavities have higher B-factors compared to the peptide present in the major binding pockets (Table 5) as well as mImpαΔIBB (average B-factor of 39.4 Å^2^; calculated by Baverage, CCP4 [71]) suggesting the TNRC6A cNLS preferentially binds to the mImpα major binding site, as described previously for other cNLS peptides.

**Table 4:** Comparison of monopartite cNLS interactions with Impα minor binding site.

cNLS	N-term	Minor binding site pockets				C-term	PDB ID
		P1’	P2’	P3’	P4’		
TNRC6A	K	K	R	R	R	E	5UMZ; this study
SV40 TAg	PK	K	K	R	K	V	1EJL [56],1BK6* [41]
SV40 TAg	KK	K	R	K	V		1Q1S [70],4B8O^ [53],4RXH^^ [59]
PepTM	K	K	R	R	E	A	3L3Q [57]
c-Myc		K	R	V	K	L	1EE4* [60]
dUTPase	PS	K	R	A	R	P	4MZ5 [61], 4MZ6 [61]
TAF8		K	K	S			4WV6** [63]
pUL56	TR	K	R	P	R		5HUY [64]
pUL15	PP	K	K	R	A		5HUW [64]
XPG1	SL	K	R	K	R		5EKF [65]

*, **, ^ and ^^denotes *S*. *cerevisiae*, human, *O*. *sativa* and *N*. *crassa* proteins, respectively. All other structures correspond to mImpα1.

**Table 5:** Analysis of TNRC6A interaction with mImpαΔIBB major and minor binding sites.

	Number cNLS residues built	Number H bonds	Number Salt bridges	Contacts < 4Å	Total BSA peptide^#^(Å^2^)	B-factor (Å^2^)
Major binding site	8	19	2	168	777	43.5
Minor binding site	6	12	2	136	704	69.7

In summary, our results demonstrate that TNRC6A binds directly to Impα with a monopartite cNLS, and suggest that the interactions are conserved between human and mouse complexes. The TNRC6A cNLS binds to mImpα1ΔIBB with moderate affinity, closely resembling the sequences of optimised peptides that have been directed towards the Impα major binding site. Apparent K_d_ measurements of TNRC6A cNLS mutants suggest that the requirements for high affinity binding of monopartite cNLSs to mImpα1ΔIBB depend on both the pattern of positively charged residues that interact with the major binding site pockets, but also on the N-terminal sequences that flank the basic cluster. Our results highlight the ability of monopartite cNLS peptides to maximise contacts with the Impα surface not only in the P1-P5 cavities, such as the salt bridge and H-bond interactions observed at the P3 and P4 positions described above, but also in regions extending outside the major binding site pockets.

**Fig 6:**
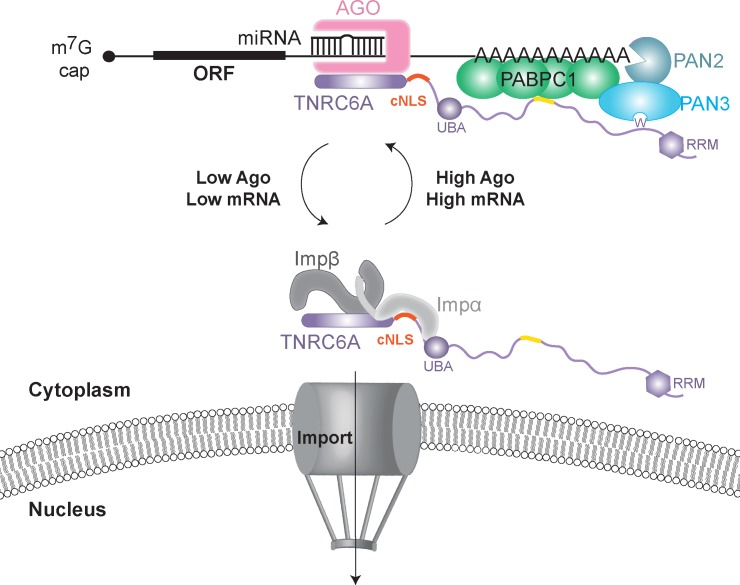
Model of regulation of TNRC6A nuclear import. TNRC6A is associated with miRISC complexes on target mRNAs in the cytoplasm via N-terminal GW motifs, while recruiting the decay machinery with C-terminal GW repeats. When not in complex with miRISC, TNRC6 is able to shuttle to the nucleus via interactions with Impα and Impβ.

In the context of the full length sequence, the Ago binding region of TNRC6A flanks the monopartite cNLS motif (Fig 1A). Although the trajectory of the cNLS mainchain N-terminal to Gly1165 is unclear from our structural work, the elongated arrangement of Impα does suggest potential steric hindrance of the binding partners, which could modulate the nuclear import activity of TNRC6A (Fig 6). Nonetheless, our results provide the basis to both enhance (N-term mutant) and impair (P2 mutant) the interaction between TNRC6A and Impα, which may be exploited for the future characterisation of TNRC6A localisation and subcellular function. Further work to identify the TNRC6A region that binds to Impβ will also assist in determining whether TNRC6 proteins have a role in mammalian cell nuclei, or whether nuclear import serves as a storage mechanism when TNRC6 proteins are not required for cytoplasmic miRNA-mediated silencing.

## Supporting information

S1 Fig TNRC6A cNLS does not interact with Impβ *in vitro*. Pull-downs with GST-NLS or GST-mImpα1 full-length with His-tagged mouse and human Impβ. GST and mImpα1 served as the negative and positive controls, respectively.
(EPS)

S2 Fig mImpα interface residues with TNRC6A cNLS are highly conserved in human proteins. Sequence alignment of mImpα1 and human Impα proteins that have been structurally determined. Residues that are >70% similarity are shown in red, strictly conserved positions are shown in white with red background. mImpα1ΔIBB residues, and the equivalent positions in the human proteins, that interface with TNRC6A cNLS are highlighted with a black frame. Residues that typically form salt bridge interactions with cNLS side chains (Asp192 and Glu396 in mImpα1) are marked with an asterisk.
(EPS)

S3 Fig Comparison of mImpα and *S*. *cerevisiae* Impα P3 binding pockets and cNLS sequences. (A) mImpα1ΔIBB is shown as grey ribbon representation, *S*. *cerevisiae* Impα1ΔIBB (PDB ID 1EE4) is shown in purple ribbon representation. TNRC6A and c-Myc cNLSs are shown as grey and purple sticks, respectively. Substitution in the *S*. *cerevisiae* P3 binding pocket (T234 is N228 in mouse) is highlighted. (B) cNLS sequences are aligned by major binding site interactions. The P2 lysine is highlighted in blue and the P1’ and P2’ positions are underlined in red.
(EPS)
